# Comparison of Mental Health Service Utilization by Asian Americans and Non-Hispanic Whites versus Their Cardiovascular Care Utilization

**DOI:** 10.7759/cureus.1595

**Published:** 2017-08-22

**Authors:** Benjamin K Woo

**Affiliations:** 1 UCLA

**Keywords:** asians, asian americans, chinese americans, ethnic disparities, mental health service, health service utilization, schizophrenia

## Abstract

Introduction: This study will determine whether racial/ethnic disparities persist in various psychiatric disorders among Asian Americans.

Methods: Secondary analyses of data from the second largest public health system in the US (total N=22294) were performed. Chi-squared statistics were used to compare the race for mental health service utilization for five psychiatric diagnoses. Cardiovascular care utilization by Asian Americans and non-Hispanic whites was used as a proxy for overall healthcare utilization rates between the two racial groups and constituted the expected values for the analysis.

Results: Asian Americans were less likely to utilize mental health services for bipolar disorder, schizophrenia, anxiety, depression, and intellectual disabilities.

Conclusion: The results of this study call for ways to increase mental health service utilization on par with cardiovascular healthcare utilization among Asian Americans.

## Introduction

Asian Americans are included as one of the major racial categories in the 2010 US Census [1]. The most recent census estimates show that there are 1.3 million Asian Americans in Los Angeles County, which accounts for 13.8% of the total county population. As this population continues to grow, healthcare providers must be cognizant of the considerable disparity in the recorded epidemiological prevalence of mental health issues and in mental health service utilization between Asian Americans and the general population [2-5].

The National Latino and Asian American Study (NLAAS) was the first national epidemiological survey of Asian Americans in the United States [6]. The study surveyed a national sample of 2095 Asian American individuals. The overall lifetime rate of Asian Americans having substance use disorders, anxiety disorders, or depression was 17.3%. Data from the National Epidemiological Survey on Alcohol and Related Conditions (NESARC) revealed that the 12-month prevalence of any psychiatric disorder was 23.6% and 22.3% for Asian American males and females, respectively [5].

Despite the substantial risk for psychiatric symptoms among Asian Americans, racial disparities have been found in mental health service utilization by this group [2,7]. Data from the NLAAS revealed Asian Americans have lower rates of mental-health-related service use compared to the general population [2]. Additionally, a lower percentage of Asian Americans (34.1%) with probable mental disorders sought any form of service as compared to their counterparts (41.1%). The NESARC study showed that only 25% of Asians with any Diagnostic and Statistical Manual of Mental Disorders - Fourth Edition (DSM-IV) psychiatric disorders utilized mental health services [7].

In the United States, the overall prevalence of hypertension among adults was 29% for the period 2011-2014 [8]. The prevalence of hypertension was lower among Asian Americans (24.9%) than among non-Hispanic whites (28.0%). In addition, the estimated prevalence of ischemic heart diseases (IHD) was lower among Asian Americans (4.3%) as compared to 6.4% of the total population [9]. Therefore, cardiovascular care utilization by Asian Americans and non-Hispanic whites may serve as a proxy denominator to estimate the race/ethnicity distribution of a service area.

It is unclear whether ethnic/racial disparities persist in various psychiatric disorders among Asian Americans. Our primary goal was to measure overall ethnic/racial disparities by comparing mental health service utilization by Asian Americans and non-Hispanic whites versus their utilization of health-related service use for primary hypertension (PH) and IHD. Our secondary goal was to measure ethnic/racial disparities by comparing the mental health service utilization of five psychiatric disorders by Asian Americans and non-Hispanic whites versus their utilization of PH health-related service use.

## Materials and methods

The study was carried out at the Los Angeles County’s Department of Health Services (LACDHS). LACDHS is the second-largest public health system in the US and serves nearly 10 million residents. LACDHS includes four traditional hospital-based facilities as well as several off-site clinics. For the purpose of analysis, data derived from the LACDHS Electronic Health Record consists of a combined pool of patient visits to inpatient, emergency, and hospital outpatient facilities. The basic sampling unit is the physician-patient encounter or visit. Study participants included individuals of Asian or non-Hispanic white race/ethnicity between 18 and 65 years of age.

Data collected over a 30-month period (January 1, 2015 – June 30, 2017) included patient demographics, diagnoses, and various chronic conditions. We examined a subset of participants who had international classification of disease, tenth revision (ICD-10) codes for one of five psychiatric diagnoses: 1) Anxiety, dissociative, stress-related, somatoform, and other nonpsychotic mental disorders (F40-F48); 2) Bipolar disorders or manic episodes (F30-F31); 3) Intellectual disabilities (F70-F79); 4) Major depressive disorders (F32-F33); and 5) Schizophrenia, schizotypal, delusional, and other non-mood psychotic disorders (F20-F29). A comparison group with primary hypertension (I10) was selected to determine disparities in mental health service utilization. The race-proportions of visits for PH were used as the expected values in the chi-squared analyses. A separate analysis was then performed using the race-proportions of visits for ischemic heart diseases (I20-I25) as a comparison group to confirm the overall ethnic/racial disparities. 

Data collected from the LACDHS electronic health record were analyzed with chi-squared statistics to compare the effect of race/ethnicity on mental health service utilization for each of the five psychiatric diagnoses with the effect of race/ethnicity on service utilization for PH. This research was deemed exempt research involving human subjects by the LACDHS, as it utilized pre-existing, de-identified data. Data were entered and analyzed using IBM SPSS Statistics v. 21.0 (Armonk, NY).

## Results

Of the 22294 psychiatric health service visits in the study, 2735 (12.3%) visits were from Asian Americans and 19559 (87.7%) visits were from non-Hispanic Whites. There were 11129 (49.9%) females and 11165 (50.1%) males. The study included 8444 (37.9%) cases aged 18-34 years old, 4399 (19.7%) cases aged 35-44 years old, 4673 (21.0%) cases aged 45-64 years old, and 4778 (21.4%) cases aged 55-64 years old. Table 1 presents descriptive statistics for health service visits relating to each of the five psychiatric diagnoses.

**Table 1:** Demographics of cases with the five psychiatric diagnoses 1) Source of Data: Los Angeles County's Department of Health Services (LACDHS) electronic health record. 2) International classification of disease, tenth revision (ICD-10) codes were provided underneath each one of the five psychiatric diagnoses.

	Schizophrenia (F20-F29)		Bipolar Disorders (F30-F31)		Depression (F32-F33)		Anxiety (F40-F48)		Intellectual Disabilities (F70-F79)		Total	
	(n=5437)		(n=2011)		(n=6629)		(n=7742)		(n=475)		(n=22294)	
Gender												
Female	1853	34.1%	977	48.6%	3726	56.2%	4356	56.3%	215	45.3%	11129	49.9%
Male	3584	65.9%	1034	51.4%	2903	43.8%	3386	43.7%	260	54.7%	11169	50.1%
Living status												
Living	5425	99.8%	2005	99.7%	6605	99.6%	7709	99.6%	471	99.2%	22215	99.6%
Deceased	12	0.2%	6	0.3%	24	0.4%	33	0.4%	4	0.8%	79	0.4%
Race/Ethnicity												
Asian	572	10.5%	185	9.2%	913	13.8%	995	12.9%	70	14.7%	2735	12.3%
Whites	4865	89.5%	1826	90.8%	5716	86.2%	6747	87.1%	405	85.3%	19559	87.7%
Age group												
18-34	2372	43.6%	768	38.2%	2067	31.2%	2952	38.1%	285	60.0%	8444	37.9%
35-44	1225	22.5%	447	22.2%	1091	16.5%	1555	20.1%	81	17.1%	4399	19.7%
45-64	1051	19.3%	442	22.0%	1544	23.3%	1567	20.2%	69	14.5%	4673	21.0%
55-64	789	14.5%	354	17.6%	1927	29.1%	1668	21.5%	40	8.4%	4778	21.4%

For PH health-related service use, there were 5473 (25.0%) Asian American cases and 16428 (75.0%) non-Hispanic white cases. Compared to non-Hispanic whites, Asian Americans were significantly less likely to utilize any mental health services, even when accounting for changes in health care utilization (using PH health-related service use as a proxy) that already exist between races (chi-square=1182.34, df=1, p<.01). This significant trend exists across the continuum of mental health disorders: Asian Americans were less likely to utilize mental health services for anxiety disorders (chi-square=494.00, df=1, p<.01), bipolar disorders (chi-square=254.25, df=1, p<.01), intellectual disabilities (chi-square=26.23, df=1, p<.01), major depressive disorders (chi-square=368.53, df=1, p<.01), and schizophrenia (chi-square=529.48, df=1, p<.01). 

For IHD health-related service use, there were 575 (22.8%) Asian American cases and 1945 (77.2%) non-Hispanic white cases. Using IHD-related utilization as a proxy to estimate the race/ethnicity distribution of the service area, Asian Americans, in comparison to non-Hispanic whites, were significantly less likely to utilize any mental health services (chi-square=217.98, df=1, p<.01).

## Discussion

The present study explored the racial disparities in the delivery of care to patients with psychiatric disorders as compared to patients with PH. Significant differences were seen in overall mental health service use, with less Asian Americans than non-Hispanic whites receiving mental health service even when compared to their respective hypertension healthcare service utilization. This finding was confirmed with IHD health-related service use as another proxy to estimate the race/ethnicity distribution of the denominator population. This pattern is consistent with previous reports on ethnic disparities between Asian Americans and non-Hispanic whites [2,7]. Nonetheless, the implications and reasons for such ethnic disparities require further inquiry to ensure an equal quality of care for vulnerable populations.

Despite comparable rates of mental health disorders and overall disease among Asian Americans and non-Hispanic whites, this study revealed that while 25.0% of hypertension health care was utilized by Asian Americans, they only accounted for 9.2%, 10.5%, 12.9%, 13.8%, and 14.7% of mental health service utilization for bipolar disorder, schizophrenia, anxiety, depression, and intellectual disabilities, respectively. Particularly for Asian Americans, stigma and other barriers can deter patients and their families from seeking needed mental health services [10-11]. In comparison with medical illnesses, the stigma surrounding psychiatric disorder contributes to shame in the Asian American community, which can affect both quality and access to care [12-13]. Our results further highlight that among psychiatric disorders, Asian Americans with schizophrenia or bipolar disorders were less likely to utilize mental health service. Innovations in delivering culturally sensitive health education are needed to reduce such disparities in health and health care [14-15].

This study has several limitations. First, the nature of pre-existing, de-identified data of one county impacts its generalizability. Second, without individual-level data, the study did not account for other factors that have shown to determine racial disparities. Third, we have no detailed information on the mental health visits. Fourth, the study did not account for racial/ethnic differences in care-seeking for PH and IHD. The observation of different race/ethnicity distribution could actually have more to do with disparities in cardiac issues than with disparities in mental health care. Future research will need to be performed to further delineate these relationships. Finally, the dataset did not distinguish among Asian American subgroups; it is important to acknowledge that Asian Americans comprise a heterogeneous group. Despite these limitations, our study calls for ways to increase mental health service utilization on par with cardiovascular healthcare utilization among Asian Americans.

## Conclusions

Asian Americans were less likely to utilize mental health services for bipolar disorder, schizophrenia, anxiety, depression, and intellectual disabilities. The results of this study call for ways to increase mental health service utilization on par with cardiovascular healthcare utilization among Asian Americans.
